# A Japanese man with spina bifida who successfully fathered a child following fertility treatment

**DOI:** 10.1002/iju5.12149

**Published:** 2020-03-07

**Authors:** Daisuke Gotoh, Katsuya Aoki, Yosuke Morizawa, Shunta Hori, Yoshitaka Itami, Makito Miyake, Kazumasa Torimoto, Hitoshi Momose, Kiyohide Fujimoto

**Affiliations:** ^1^ Department of Urology Nara Medical University Kashihara Nara Japan; ^2^ Department of Urology Hirao Hospital Kashihara Nara Japan

**Keywords:** assisted reproductive technology, conception, fertility treatment, intracytoplasmic sperm injection, spina bifida

## Abstract

**Introduction:**

Spina bifida is a congenital anomaly caused by a neural tube closure defect that may result in sexual dysfunction. Sexual dysfunction and infertility are prevalent in spina bifida patients but have been scarcely reported.

**Case presentation:**

We report the case of a 36‐year‐old man with spina bifida and mild‐moderate erectile dysfunction. He could experience erection and ejaculation. He got married at the age of 28 years, but his wife was unable to conceive for 2 years thereafter. Semen analyses revealed that semen volume, sperm density, and sperm motility rate were below normal levels. It was concluded that natural conception would be difficult, and assisted reproductive strategies were planned. After 4 years, his wife conceived through intracytoplasmic sperm injection and gave birth to a healthy baby.

**Conclusion:**

Fertility treatment, including intracytoplasmic sperm injection, is a useful therapeutic method for male patients with spina bifida who desire to father a child.

**Abbreviations & Acronyms**
ART: assisted reproductive technology
CIC: clean intermittent catheterization
ICSI: intracytoplasmic sperm injection
IVF: in vitro fertilization
QOL: quality of life
SHIM: Sexual Health Inventory for Men

**Keynote message**

The subject of this case report is a 36‐year‐old man with spina bifida. Following marriage at the age of 28, his wife was unable to conceive for 2 years. Semen analyses showed that semen volume, sperm density, and sperm motility rate were below normal levels. After 4 years of assisted reproduction, his wife conceived through ICSI and gave birth to a healthy baby. ICSI is a useful therapeutic method for male patients with spina bifida who intend to have children.

## Introduction

Spina bifida is a congenital anomaly caused by a neural tube closure defect that may result in lower limb, lower urinary tract and bowel, and sexual dysfunction. Individuals with spina bifida require a combination of treatment approaches, including neurosurgery, orthopedic treatment, urological treatment, and a lifetime of rehabilitation.

Medical and surgical advances in spina bifida treatment and management, particularly the introduction of CIC,^1^ have improved lower urinary tract passage disorder and decreased the frequency of renal function disorder,^2^, ^3^ but issues related to sexual function and fertility in men remain.

Sexual dysfunction and infertility are prevalent in spina bifida patients, but only a few reports exist regarding this condition.

We describe a man with spina bifida who received fertility treatment and could father a child.

## Case presentation

A 36‐year‐old man was born with a spinal cord lipoma at the L5‐S1 level. Soon after birth, he underwent surgery for spinal lipoma removal. He could walk whilst dragging the left foot, and he performed bladder expression (Credé maneuver) during childhood. The modality of urination was changed to CIC at the age of 10 years, owing to vesicoureteral reflux. He underwent cystometrography at 27 years of age: his bladder compliance was as low as 9.0 mL/cmH_2_O with a capacity of 100 mL. Anticholinergic agent administration was initiated. Consequently, his bladder capacity increased to 200 mL, bladder compliance improved, and urination interval lengthened. Regarding evacuation, he started transanal irrigation at the age of 27 years, and his QOL improved. He could experience erection and ejaculation. His SHIM scores were 3‐3‐3‐2‐2, indicating mild‐to‐moderate erectile dysfunction, and his force of ejaculation was weak.

**Figure 1:**
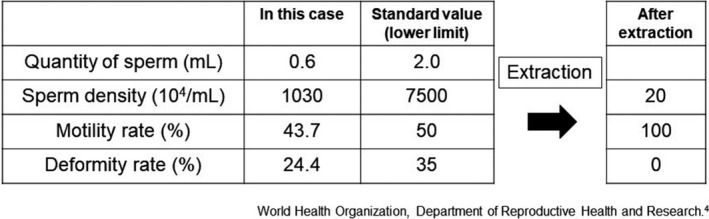
Sperm findings. After sperm are retrieved using a microscope, high quality sperm are isolated. WHO laboratory manual for the examination of human semen 5th edn.^4^

He got married at the age of 28 years but failed to father a child for 2 years thereafter. Consultation with a fertility specialist and sperm examination showed a sperm quantity of 0.6 mL, density 10 300 000/mL, and motility rate 43.7%; all three parameters being lower than normal according to the World Health Organization criteria.^4^ Furthermore, the sperm deformity rate was 24.4% (Fig. 1). The sizes of the patient’s right and left testes were 11.7 and 7.3 cm^3^, respectively. The sizes are small compared to the standard. It was concluded that natural conception would be difficult; thus, the patient underwent several rounds of ICSI. After undergoing fertility treatment for 4 years, his wife conceived and gave birth to a healthy baby.

## Discussion

Spina bifida patients face various problems related to activities of daily living, including walking and urination, and may require nursing care in the presence of hydrocephalus. The QOL of spina bifida patients improved significantly after Lapides *et al*. introduced a CIC technique for lower urinary tract dysfunction in 1972.^1^ Moreover, further improvements have been made with advances in bowel dysfunction management and spinal column and lower limb transformation techniques. With these recent improvements, medical personnel and researchers have focused on the QOL of spina bifida patients.^5^

Fertility in spina bifida patients has attracted attention recently, and 80–100% of post‐pubertal male spina bifida patients have normal sexual desires.^6^ Thus, one of the most important issues in spina bifida is to address the cause of male infertility. Poor sperm quality due to testicular or ejaculation dysfunction is the most important factor in this patient’s infertility. For testicular dysfunction, there are few reported cases on spina bifida, although studies on patients with spinal cord injury exist. In these patients, when the period post‐injury exceeds 12 years, it becomes difficult to collect sperm.^7^ Moreover, when these patients experience repeated UTIs such as epididymitis, disorders in the passage of sperm could occur. However, information about fertility treatment for these patients is insufficient. A study of 57 men with spina bifida found that of the 11 (19%) patients who attempted to father children, only eight (14%) were successful.^8^ Thus, many such patients may believe that fertility treatment is not available for them, owing to the lack of information and awareness regarding suitable centers equipped with the required resources. Moreover, information about sexual dysfunction and fertility treatment is not readily available even for the general population, and the same can be said for spina bifida patients.

The risk of inheriting a neural tube closure disorder is 4% when either parent has spina bifida; this risk is 7.7% among female and 2% among male infants.^9^ However, this risk is considered to decrease with folic acid intake during pregnancy.^10^ In the present case, the patient’s wife was administered a folate supplement during the fertility treatment.

**Figure 2:**
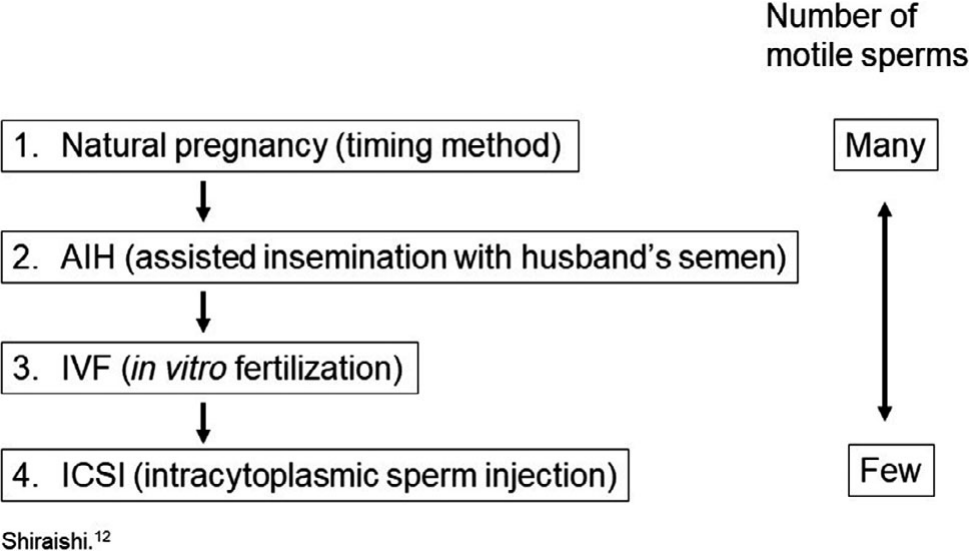
Course of infertility treatment. IVF and ICSI are implemented to increase the number of motile sperm.^12^

Methods of conception can be classified into four types as follows: natural pregnancy, assisted insemination with the husband’s semen, IVF, and ICSI. IVF and embryo transfer have developed as ART treatments since the conception of the first child via ART in 1978.^11^ Generally, IVF and ICSI are recommended for cases of insufficient ejaculated sperm volume^12^ (Fig. 2). However, because the quality of the sperm was not satisfactory in our case, ICSI was performed from the outset. ICSI is recommended when natural pregnancy is considered impossible or notably difficult owing to male sterility and fertilization disorders. Moreover, because erectile dysfunction and ejaculation disorders are common among spinal cord injury patients, ICSI may prove to be very useful in such cases.^13^

Reports on male spina bifida patients fathering children following fertility treatment are rare; thus, findings from our case are a valuable contribution to literature. However, fertility treatment is not without its challenges. First, the physical and mental burden on the female partner is significant, and it is important to counsel her adequately throughout the process. Second, fertility treatment is cost‐intensive, which proves to be a challenge for many couples because it is not covered by insurance in Japan. In addition, it is difficult for couples to ascertain when fertility treatment should be stopped due to a lack of response. Thus, considering the mental and physical burden and high cost of ART, it is important that couples take their time to make an informed decision.

## Conclusion

For male patients with spina bifida who desire to father a child, despite the many dysfunctions that make conception challenging with this condition, fertility treatment, including ICSI, is a useful therapeutic method.

## Ethical statement

The protocol for this research project has been approved by a suitably constituted Ethics Committee of the institution and it conforms to the provisions of the Declaration of Helsinki. All human subjects provided written informed consent with guarantees of confidentiality.

## Conflict of interest

The authors declare no conflict of interest.

## References

[1] Lapides J , Diokno AC , Silber SJ , Lowe BS . Clean, intermittent self‐catheterization in the treatment of urinary tract disease. J. Urol. 1972; 107: 458–61. 5010715 10.1016/s0022-5347(17)61055-3

[2] Obara K , Mizusawa T , Isahaya E , Suzuki K , Hara N , Takahashi K . Efficacy of clean intermittent catheterization for urinary incontinence in children with neurogenic bladder dysfunction secondary to myelodysplasia. Low. Urin. Tract Symptoms 2010; 2: 100–5. 26676291 10.1111/j.1757-5672.2010.00070.x

[3] Veenboer PW , Bosch JL , van Asbeck FW , de Kort LM . Upper and lower urinary tract outcomes in adult myelomeningocele patients: a systematic review. PLoS One 2012; 7: e48399. 23119003 10.1371/journal.pone.0048399 PMC3485227

[4] World Health Organization, Department of Reproductive Health and Research . WHO laboratory manual for the examination of human semen, 5th edn (Internet). 2010 [Cited 30 Jun 2019.]. Available from URL: http://www.who.int/reproductivehealth/publications/infertility/9789241547789/en/

[5] Deng N , Thirumavalavan N , Beilan JA *et al* Sexual dysfunction and infertility in the male spina bifida patient. Transl. Androl. Urol. 2018; 7: 941–9. 30505732 10.21037/tau.2018.10.08 PMC6256049

[6] Dorner S . Sexual interest and activity in adolescents with spina bifida. J. Child Psychol. Psychiatry 1977; 18: 229–37. 330550 10.1111/j.1469-7610.1977.tb00435.x

[7] Iwahata T , Shin T , Shimomura Y *et al* Testicular sperm extraction for patients with spinal cord injury‐related anejaculation: a single‐center experience. Int. J. Urol. 2016; 12: 1024–7. 10.1111/iju.13226 27766729

[8] Decter RM , Furness PD 3rd , Nguyen TA , McGowan M , Laudermilch C , Telenko A . Reproductive understanding, sexual functioning and testosterone levels in men with spina bifida. J. Urol. 1997; 157: 1466–8. 9120984

[9] Kadir RA , Sabin C , Whitlow B , Brockbank E , Economides D . Neural tube defects and periconceptional folic acid in England and Wales: retrospective study. BMJ 1999; 319: 92–3. 10398632 10.1136/bmj.319.7202.92 PMC28158

[10] MRC Vitamin Study Research Group . Prevention of neural tube defects: results of the Medical Research Council Vitamin Study. Lancet 1991; 338: 131–7. 1677062

[11] Dow K . “The men who made the breakthrough:” How the British press represented Patrick Steptoe and Robert Edwards in 1978. Reprod. Biomed. Soc. Online 2017; 4: 59–67. 29774267 10.1016/j.rbms.2017.07.002 PMC5952836

[12] Shiraishi K . Sexual dysfunction and male infertility in men with spina bifida. Nerv. Syst. Child. 2016; 41: 216–22.

[13] Trofimenko V , Hotaling JM . Fertility treatment in spinal cord injury and other neurologic disease. Transl. Androl. Urol. 2016; 5: 102–16. 26904416 10.3978/j.issn.2223-4683.2015.12.10 PMC4739989

